# Antioxidants Promote Intestinal Tumor Progression in Mice

**DOI:** 10.3390/antiox10020241

**Published:** 2021-02-04

**Authors:** Zhiyuan V. Zou, Kristell Le Gal, Ahmed E. El Zowalaty, Lara E. Pehlivanoglu, Viktor Garellick, Nadia Gul, Mohamed X. Ibrahim, Per-Olof Bergh, Marcus Henricsson, Clotilde Wiel, Levent M. Akyürek, Martin O. Bergo, Volkan I. Sayin, Per Lindahl

**Affiliations:** 1 Wallenberg Laboratory, Institute of Medicine, University of Gothenburg, 405 30 Gothenburg, Sweden; zhiyuan.zou@icloud.com (Z.V.Z.); larapehlivanoglu@gmail.com (L.E.P.); viktor.garellick@wlab.gu.se (V.G.); nadia.gul@wlab.gu.se (N.G.); Per-Olof.Bergh@wlab.gu.se (P.-O.B.); marcus.henricsson@wlab.gu.se (M.H.); 2 Sahlgrenska Center for Cancer Research, Department of Surgery, Institute of Clinical Sciences, University of Gothenburg, 405 30 Gothenburg, Sweden; kristell.le.gal.beneroso@gu.se (K.L.G.); ahmed.el.zowalaty@gu.se (A.E.E.Z.); mohamed.ibrahim@gu.se (M.X.I.); clotilde.wiel@gu.se (C.W.); 3 Wallenberg Centre for Molecular and Translational Medicine, University of Gothenburg, 405 30 Gothenburg, Sweden; 4 Department of Pathology, Institute of Biomedicine, University of Gothenburg, 405 30 Gothenburg, Sweden; levent.akyurek@gu.se; 5 Department of Biosciences and Nutrition, Karolinska Institute, 141 83 Huddinge, Sweden; martin.bergo@ki.se; 6 Department of Biochemistry, Institute of Biomedicine, University of Gothenburg, 405 30 Gothenburg, Sweden

**Keywords:** dietary antioxidants, intestinal tumors, tumor progression, inflammation

## Abstract

Dietary antioxidants and supplements are widely used to protect against cancer, even though it is now clear that antioxidants can promote tumor progression by helping cancer cells to overcome barriers of oxidative stress. Although recent studies have, in great detail, explored the role of antioxidants in lung and skin tumors driven by RAS and RAF mutations, little is known about the impact of antioxidant supplementation on other cancers, including Wnt-driven tumors originating from the gut. Here, we show that supplementation with the antioxidants N-acetylcysteine (NAC) and vitamin E promotes intestinal tumor progression in the ApcMin mouse model for familial adenomatous polyposis, a hereditary form of colorectal cancer, driven by Wnt signaling. Both antioxidants increased tumor size in early neoplasias and tumor grades in more advanced lesions without any impact on tumor initiation. Importantly, NAC treatment accelerated tumor progression at plasma concentrations comparable to those obtained in human subjects after prescription doses of the drug. These results demonstrate that antioxidants play an important role in the progression of intestinal tumors, which may have implications for patients with or predisposed to colorectal cancer.

## 1. Introduction

Accumulating evidence shows that dietary antioxidants have adverse effects on certain cancers. In large randomized clinical trials, β-carotene or vitamin E (VitE) increased the risk of lung and prostate cancer [1,2,3,4], and in mouse cancer models, n-acetylcysteine (NAC) or VitE accelerated the progression of lung and skin tumors [5,6,7,8]. Endogenous antioxidants appear to promote cancer development in a similar manner: lung cancer cells accumulate somatic mutations in KEAP1 and NFE2L2 that increase the production of endogenous antioxidants [9,10,11,12,13]. These and other studies stand in stark contrast to the decades-long notion of antioxidants having protective effects in cancer [14,15]. The conflicting results may reflect the complexity of redox cell biology and put the naïve expectations of a uniform antioxidant response to rest. Nevertheless, to identify the conditions under which dietary antioxidants pose a risk is vital given the widespread use of antioxidant supplements.

The majority of studies showing adverse effects of dietary antioxidants have investigated lung and skin tumors [1,4,5,6,7,8]. Why tumors in these organs might be extra sensitive to antioxidants is not clear. One hypothesis is that the high oxygen concentration in the breathed air fuels the generation of reactive oxygen species (ROS) in lung tumors, and that oxidative stress becomes a barrier that tumor cells must overcome to progress. Skin tumors are similarly exposed to high concentrations of oxygen. The genetic makeup of the tumor may also play a role since mouse models showing adverse effects of dietary antioxidants are mainly driven by mutations in the RAS–RAF pathway [5,6,7,8]. One important question is whether dietary and pharmacological antioxidants have adverse effects on tumors in other organs that are driven by non-RAS mutations.

Colorectal cancer has attracted considerable interest in the dietary antioxidant field. While epidemiological studies show that food rich in antioxidants such as selenium, vitamins C and E, and lycopenes is associated with a lower risk of colorectal cancer [16,17], clinical trials of dietary antioxidants have failed to show any significant effects [18]. Thus, whether dietary antioxidants have beneficial or adverse effects on colorectal cancer remains an open question.

Mutations in the APC locus are frequent early events in colorectal cancer, followed by mutations in TP53, KRAS, PiK3CA and others [19,20]. Indeed, at least 96% of colorectal cancers have aberrant WNT signaling as a consequence of genomic alterations in the APC or CTNNB1 (gene encoding β-catenin) locus [21]. Here, we used the *APC^Min/+^* mouse model for familial adenomatous polyposis, a heritable form of colorectal cancer, and human colorectal cancer cell lines to investigate the impact of dietary and pharmacological antioxidants on the initiation and progression of intestinal tumors.

## 2. Materials and Methods

### 2.1. ApcMin Mouse Model for Familial Adenomatous Polyposis

*APC^Min/+^*mice [19] were obtained from The Jackson Laboratory and kept on a C57BL/6J genetic background (JAX stock #002020). The mice were kept on a 12 h light–dark cycle (dark from 18.00 to 06.00) in a room kept at 19–21 °C with 40–70% relative humidity. The mice had an unlimited supply of food and water. All the animal procedures used in this study were approved by The Animal Research Ethics Committee in Gothenburg with Ethical Approval number 310-2012.

### 2.2. Antioxidant Treatment

A dose of 160 mg/kg of body weight of NAC (A7250, CAS no. 616-91-1, ≥99% purity, Sigma-Aldrich, Darmstadt, Germany) was administered in the drinking water (1 g/liter), calculated on the basis of the observed water intake of adult mice [7]. This equals a daily intake of 13 mg/kg of body weight in humans according to the body surface area normalization method for dose conversion [22]. Additionally, 41.3 mg/kg of body weight of vitamin E (dl-α-tocopheryl acetate; 7695–91-2, Zhejiang Medicine Co.) was administered in R34 chow pellets (Lantmännen, Stockholm, Sweden) at 0.5 g/kg of chow, calculated on the basis of observed daily food intake [7] and adjusted for inactive stereoisomers in dl-α-tocopheryl acetate [23]. This equals a daily intake of 3.4 mg/kg of body weight in humans, calculated on the basis of the body surface area normalization method, and is 10–20-fold above the recommended dietary intake of vitamin E, based on recommendations from the European Food Safety Authority (population reference intake: 11 mg/day) or the US National Academy of Medicine (recommended dietary allowance: 15 mg/day) and an average body weight of 60 kg. To assess the impact of antioxidants on the early stages of tumor development, pregnant females were treated with vitamin E or NAC from 2 weeks before birth until weaning, after which the pups were treated until 12 weeks of age. To assess the impact of antioxidants on the later stages of tumor progression, mice were administered antioxidants from the age of 12 weeks up to 19 weeks.

### 2.3. Plasma Concentrations of NAC

The levels of NAC in the mouse plasma were measured using reversed-phase liquid chromatography tandem mass spectrometry as described previously [24]. Briefly, 50 µL of dithiothreitol (300 mmol/L in water, Sigma-Aldrich, Darmstadt, Germany) and 50 µL of deuterated internal standard solution (Acetylcysteine-13C2, Sigma Aldrich, 1.12 µmol/L in methanol:water, 10: 90, *v*:*v*) were added to 50 µL of mouse plasma and left standing at room temperature for 5 min. Then, 50 µL of a TCA solution (10% trichloroacetic acid in water, *v*:*v*) was added, and the samples were vortexed for 5 min at 1500 rpm, followed by 5 min of centrifugation at 20,000 g. The supernatant was transferred to 96-well injection plates. Samples were analyzed using a Waters Acquity UPLC system with a BEH C18 analytical column (100 × 2.1 mm, 1.7 µm, Waters) coupled to a Xevo TQ XS tandem mass spectrometer (Waters) operated in positive electrospray ionization mode. The mobile phase (0.02% formic acid in water:acetonitrile (80:20, *v*:*v*)) flow rate was held at 0.3 mL/min during the whole runtime of 10 min. The column temperature and the temperature of the auto sampler were set to 30 and 10 °C, respectively. The mass spectrometer parameters were optimized for the detection of N-acetylcysteine and N-acetylcysteine-13C2 and set to capillary: 1.00 kV; desolvation temperature: 600 °C; desolvation gas flow: 1000 L/h; cone gas flow: 150 L/h; nebulizer gas pressure: 7.0 bar; cone voltage: (V); and collision energy (eV): N-acetylcysteine and N-acetylcysteine-13C2, 20 V and 10 eV. For each analyte, the most intense precursor/product ion transition was selected. Consequently, the MRM transitions used were N-acetylcysteine m/z 164 > 122, and N-acetylcysteine-13C2 166.1 > 123.1. The dwell time was 44 ms for all the analytes.

### 2.4. Tumor Multiplicity and Area

Mice were dissected, and the intestines were removed and separated into three segments: the colon and two segments of the small intestines. The segments were rinsed in PBS and prepared using a modified Swiss roll technique. The tissues were fixed in 4% formaldehyde and imbedded in paraffin, and 4 µm thin sections were stained with hematoxylin and eosin. Tumor counts and area calculations were performed on one single sagittal section from each segment by an investigator blinded to the treatment group as before [25].

### 2.5. Histopathological Assessment of Tumor Grades

In entire intestinal resections, all tubular, villous or tubulovillous adenomas were reviewed for epithelial cells regarding cell polarity, variations in nuclear size and glandular appearance. Dysplasia was graded as low when the stratification of the nuclei did not reach the luminal surface, apical mucin was present, the nuclei were elongated and dysplastic, and the mitotic activity and loss of polarity were minimal. Dysplasia was graded as high when an increased number of epithelial cells with enlarged nuclei and nuclear hyperchromatism with prominent nucleoli covering the entire epithelium from the basal layer to the apical surface, architectural changes, prominent mitotic figures, and reduced mucin were observed. Sessile serrated lesions with a sawtooth appearance of the epithelium with abundant mucin were graded with or without dysplasia. The histological assessments were performed by two observers, independently, who were blinded to the treatment group.

### 2.6. Histological Analyses and Cell Proliferation

For routine histology, 4 μm sections of paraformaldehyde-fixed, paraffin-embedded, Swiss-rolled intestines were stained with haematoxylin and eosin. For immunohistochemical analyses, sections pretreated with citrate buffer at 95 °C, Peroxidazed (Biocare Medical, Pacheco, CA, USA), and Background Sniper (Biocare Medical, Pacheco, CA, USA) were incubated with antibodies recognizing β-catenin (BD, 610154), Ki67 (RTU, RM-9106-R7, Thermo Scientific) or pH3 (ser10) (ref. 9701S, Cell Signaling, Danvers, MA, USA), and then processed with the Vectastain Elite ABC Kit (PK6101) and the DAB Peroxidase Substrate Kit (SK4100, Vector Laboratories), or MACH 1 Universal HRP-Polymer Detection Kit and Betazoid DAB. The slides were stained with an intelliPATH flx automated staining system (Biocare Medical, Pacheco, CA, USA) and scanned with a MIRAX SCAN microscope with the MIRAX Control software (Zeiss, Oberkochen, Germany). Proliferating cells were quantified with the TissueMorph software and the TISSUEalign module (Visiopharm Integrator System version 5.0.2.1158, Hoersholm, Denmark) or the Analyze Particles function in ImageJ (version 1.8.0_172).

### 2.7. Cell Culture and Analyses of Proliferation

Human colon cancer cell lines (American Type Culture Collection, ATCC) were cultured in RPMI (Colo320) or F12K (LoVo) medium supplemented with 10% fetal bovine serum (FBS) (Cat.no. 26600023, 21127022, 21875059 and 11415049, Thermo Fisher). NAC (≥99% purity; A7250) and Trolox, a water-soluble analogue of vitamin E (6-hydroxy-2,5,7,8-tetramethylchromane-2-carboxylic acid; 53101-49-8, Sigma-Aldrich, Darmstadt, Germany), were used at concentrations of 200 µM in the case of NAC and 20 µM for Trolox. Water was used as a vehicle control for NAC; DMSO was the control used for Trolox. Real-time analysis of proliferation was performed by seeding 1 × 10^4^ cells per well in 96-well ImageLock plates (Cat.no. 4379, Essen Biosciences, Ann Arbor, MI, USA) with control medium or medium supplemented with NAC or Trolox; the plates were then monitored every 1–2 h with the Incucyte System (Essen Biosciences, Ann Arbor, MI, USA) for a total of 91–106 h.

### 2.8. Redox status of Colon Cancer Lines

Colon cancer cells (LoVo and Colo320) were seeded in 96-well plates (TC-treated Corning black with transparent flat bottom). The seeding density was 25 × 10^3^ cells per well, in 100 μL of medium with or without antioxidants (200 μM NAC or 20 μM Trolox). The control for NAC was H_2_O, and DMSO was chosen for Trolox. The cells were incubated for 48 h with antioxidants and then stained with a multiprobe redox assay kit (KP-06-005, BioQuoChem, Asturias, Spain) and imaged with the Operetta HCS microscope (Perkin-Elmer, Waltham, MA, USA).

### 2.9. Statistical Analyses

Statistics were performed with non-parametric ANOVA tests with Dunn’s correction for tumor multiplicity and tumor area, chi square tests for tumor distribution, and two-way ANOVA tests with Tukey’s correction for cell growth. The GraphPad Prism software (versions 7.02 and 8.1; San Diego, CA, USA) was used for the statistical analyses, and the values are presented as mean ± SEM, unless otherwise indicated. The investigators were blinded to the treatment. A *p*-value < 0.05 was considered statistically significant.

## 3. Results

**Figure 1:**
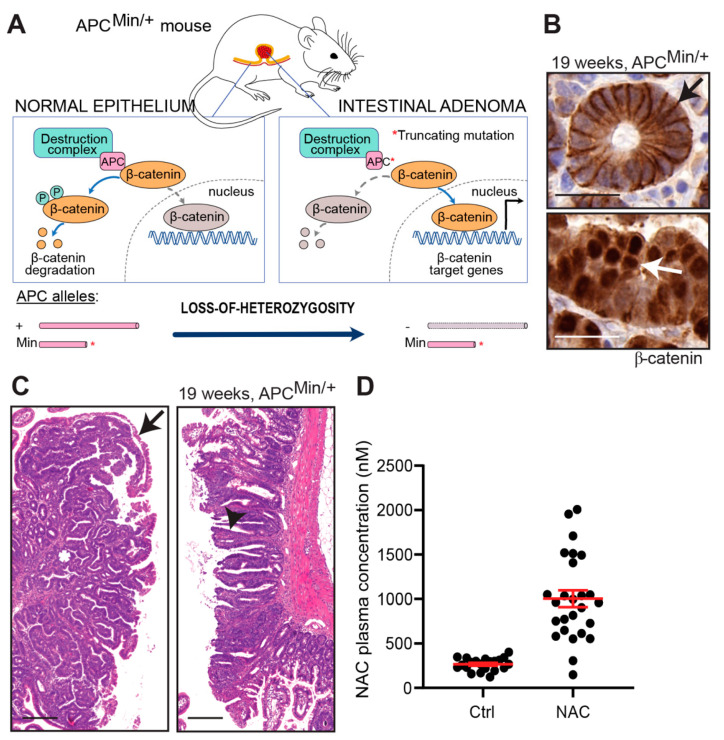
N-acetylcysteine (NAC) levels in the ApcMin model. (**A**) Schematic showing how mutant APC protein (after loss of the second *Apc* allele) cripples the destruction complex and thus abolishes phosphorylation and subsequent degradation of β-catenin. Instead, unphosphorylated β-catenin translocates to the nucleus and activates the transcription of β-catenin target genes. (**B**) Photomicrographs showing β-catenin staining in plasma membranes of normal epithelial cells (top panel, black arrow) and nuclear β-catenin staining of tumor cells (bottom panel, white arrow). (**C**) Adenomas with tubular (left) or tubulovillous (right) architecture. Asterisk indicates crypt, white arrow indicates villous, and black arrow indicates a thin layer of normal epithelium that covers tubular adenomas in a manner typical for the model. (**D**) Plasma concentrations of NAC in adult *APC^Min/+^* mice 14 days after treatment start (*n* = 21 and 26, respectively). Scale bars are 25 μm in (**B**) and 100 μm in (**C**); error bars indicate SEM.

To investigate the impact of antioxidants on intestinal tumor initiation and progression, we used the *APC^Min/+^* mouse model for familial adenomatous polyposis, a heritable form of colorectal cancer [26,27]. Tumors in *APC^Min/+^* mice are driven by the homozygous loss of the tumor suppressor APC: the first allele is inactivated by a truncating germline mutation, and the second allele, by a stochastic loss-of-heterozygosity in somatic cells, leading to constitutive WNT signaling and neoplastic transformation (Figure 1A). Because similar APC mutations are found in the majority of spontaneous human colorectal cancers, *APC^Min/+^* mice are used as a general model for the early stages of colorectal cancer. In a fashion typical for the model [26,27,28], the *APC^Min/+^* mice developed multiple intestinal neoplasia expressing nuclear β-catenin (Figure 1B) and spanning in size from aberrant crypt foci to adenomas with tubular or tubulovillous architecture (Figure 1C). The tumors were mainly located in the distal small intestine and did not invade the submucosa or metastasize to lymph nodes or distant organs.

The mice were given chow supplemented with VitE (0.5 g/kg) at a concentration 15-fold above the daily requirement of the mouse [29] or drinking water supplemented with NAC (1 g/L). Control mice were given regular chow with the recommended daily intake of VitE and regular drinking water. Because mice metabolize drugs faster than humans, we used a NAC dosage that was higher than those recommended for humans without being toxic to the mice. To verify that the dosage was adequate, we measured the NAC concentration in the plasma 14 days after the treatment started in the adult mice. The concentration was 1.0 µM (95% CI: 0.81–1.2 µM) in the NAC-treated mice and 0.27 µM (95% CI: 0.23–0.30 µM) in the controls (Figure 1D). Thus, the plasma concentration of NAC in the *APC^Min/+^* mice was below or comparable to the concentrations measured in human subjects that were given prescription doses of NAC [30].

**Figure 2:**
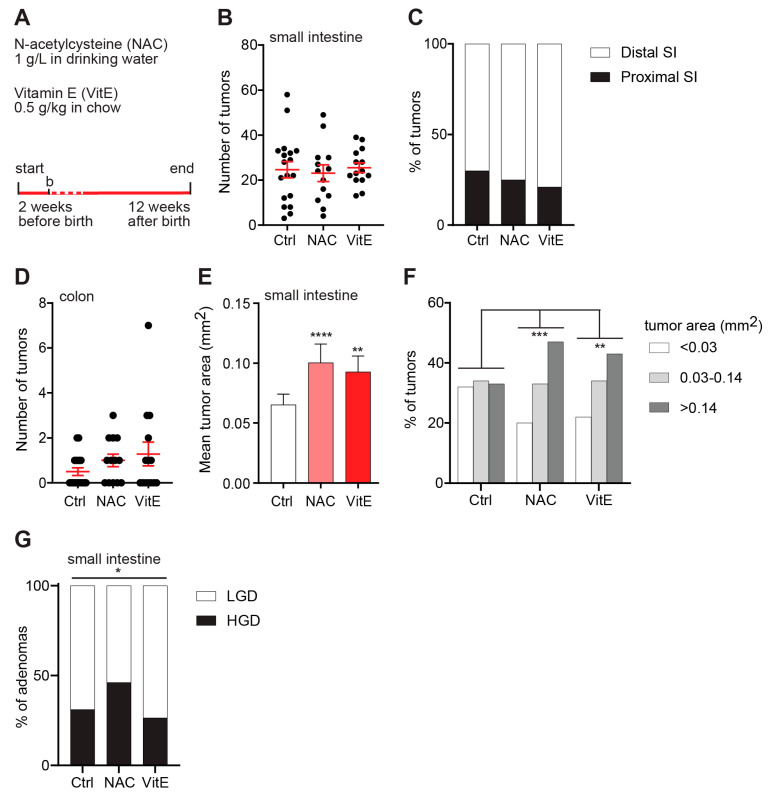
NAC or vitamin-E treatment increases the size of early tumors. (**A**) Schematic of the study design. Two structurally unrelated dietary antioxidants, NAC and VitE, were supplemented in the drinking water or chow diet of pregnant dams from two weeks before birth until weaning and, thereafter, to the pups until 12 weeks of age. Dashed line indicates breast feeding period. (**B**,**C**) Tumor numbers in one sagittal section of the small intestine (**B**) and the distribution of tumors between the proximal and distal parts of the small intestine (**C**) of 12-week-old *APC^Min/+^* mice (*n* = 18 Ctrl, 13 NAC, 14 VitE). (**D**) Number of colonic tumors in 12-week-old *APC^Min/+^* mice (*n* = 18 Ctrl, 13 NAC, 14 VitE). (**E**,**F**) The geometric mean area (**E**) and size distribution (**F**) of tumors in the small intestines of 12-week-old *APC^Min/+^* mice (*n* = 303-427 tumors, error bars indicate 95% confidence intervals). (**G**) Distribution graph showing the percentage of adenomas with HGD or LGD in the small intestines of 12-week-old *APC^Min/+^* mice (*n* = 74-102 adenomas per group). HGD, high-grade dysplasia; LGD, low-grade dysplasia. * *p* < 0.05, ** *p* < 0.01, *** *p* < 0.001, and **** *p* < 0.0001.

To assess the impact of antioxidants on the early stages of tumor development, pregnant dams were treated with VitE or NAC from 2 weeks before birth until weaning, after which the pups were treated until 12 weeks of age (Figure 2A). The intestines were prepared as Swiss rolls and investigated under the microscope. There was no difference in the numbers of intestinal or colonic tumors between the NAC- or VitE-treated *APC^Min/+^* mice and controls, and there was no difference in the distribution of tumors between the proximal and distal parts of the small intestine (Figure 2B,C). However, the tumors in the NAC- and VitE-treated *APC^Min/+^* mice were markedly larger compared to untreated controls (Figure 2D). The size distribution revealed a smooth transition from small and medium-sized tumors towards larger tumors (Figure 2E). Moreover, histopathological classification revealed a small but significant increase in adenomas with high-grade dysplasia (HGD) in the NAC-treated *APC^Min/+^* mice compared to controls (Figure 2F). There was no increase in HGD adenomas in the VitE-treated *APC^Min/+^* mice at this time point (Figure 2F).

**Figure 3:**
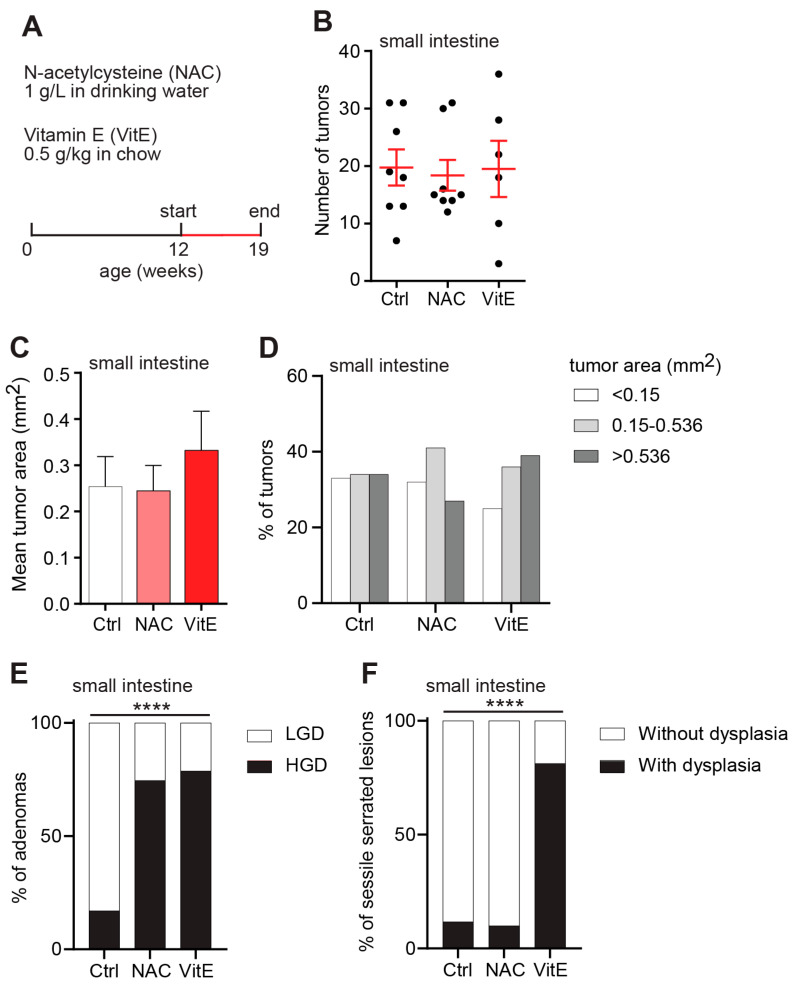
NAC or vitamin-E treatment increases the histopathological grade of advanced tumors**.** (**A**) Schematic of the study design. NAC or VitE were supplemented in the drinking water or chow diet of *APC^Min/+^* mice from 12 until 19 weeks of age. (**B**) Tumor numbers in one sagittal section of the small intestine of 19-week-old *APC^Min/+^* mice (*n* = 8 Ctrl, 8 NAC, 6 VitE). (**C**,**D**) The geometric mean area (**C**) and size distribution (**D**) of individual tumors in the small intestines of 19-week-old *APC^Min/+^* mice (*n* = 117–158 tumors, error bars indicate 95% confidence intervals). (**E**,**F**) Distribution graphs showing the percentage of adenomas with HGD or LGD (E), or sessile serrated lesions (SSLs) with or without dysplasia (**F**), in the small intestines of 19-week-old *APC^Min/+^* mice (*n* = 47–67 adenomas, 10-17 SSLs). HGD, high-grade dysplasia; LGD, low-grade dysplasia. **** *p* < 0.0001.

A second cohort of *APC^Min/+^* mice was treated with antioxidants between weeks 12 and 19 postpartum to assess the impact on more advanced tumors (Figure 3A). In line with our previous results, there was no difference in tumor counts between the NAC- or VitE-treated *APC^Min/+^* mice and controls (Figure 3B). Neither could we detect any difference in tumor size between the treatment groups at this age (Figure 3C). By contrast, NAC and VitE had dramatic effects on tumor grades. The number of adenomas with HGD was markedly increased in the NAC- and VitE-treated *APC^Min/+^* mice compared to controls (Figure 3D). The dysplastic features included enlarged nuclei, nuclear hyperchromatism, cribriform crypts, prominent mitotic figures, and reduced mucin. Moreover, the VitE-treated *APC^Min/+^* mice showed increased proportions of sessile serrated lesions (SSLs) with dysplasia compared to control (Figure 3E,F). These lesions are associated with a high risk of malignant transformation [31].

**Figure 4:**
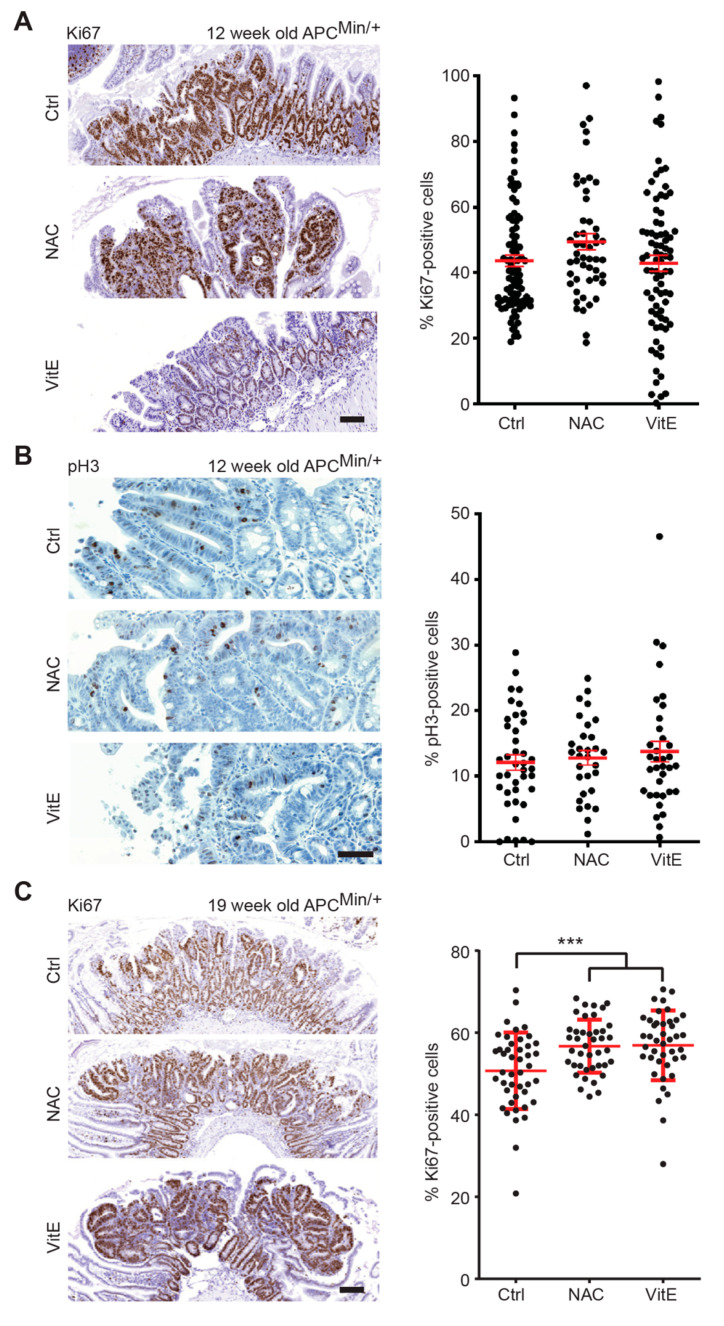
NAC and VitE increase tumor cell proliferation at 19 weeks of age. (**A**,**B**) Immunohistochemical staining of Ki67 (**A**) or phosphorylated histone 3 (pH3) (**B**) in adenomas from small intestines of *APC^Min/+^* mice that were treated with NAC or VitE from two weeks before birth until 12 weeks of age or untreated controls. Antigen and treatment are indicated in the panels. Dot plots showing percent Ki67 or pH3-stained cells in individual tumors (*n* = 50–90 tumors/group in **A**; *n* = 30–40 tumors/group in **B**). (**C**) Immunohistochemical staining of Ki67 in adenomas from small intestines of *APC^Min/+^* mice that were treated with NAC or VitE from 12 to 19 weeks of age or untreated controls. Antigen and treatment are indicated in the panels. Dot plots showing percent Ki67 or pH3-stained cells in individual tumors (*n* = 42-44 tumors/group). Scale bars are 10 μm in (**A**,**C**) and 50 μm in (**B**); error bars indicate SEM; *** *p* < 0.001.

Antioxidant treatment has, in earlier studies, increased tumor cell proliferation in mice [7]. We therefore investigated intestinal tumors at week 12 (early treatment) and week 19 (late treatment) for the expression of the proliferation markers Ki67 or phosphorylated histone 3. There was no difference in tumor cell proliferation between the antioxidant-treated *APC^Min/+^* mice and controls in the early treatment group (Figure 4A,B). However, NAC or VitE increased the rate of proliferating cells in the late treatment group (Figure 4C). Thus, antioxidants can increase intestinal tumor cell proliferation in *APC^Min/+^* mice.

**Figure 5:**
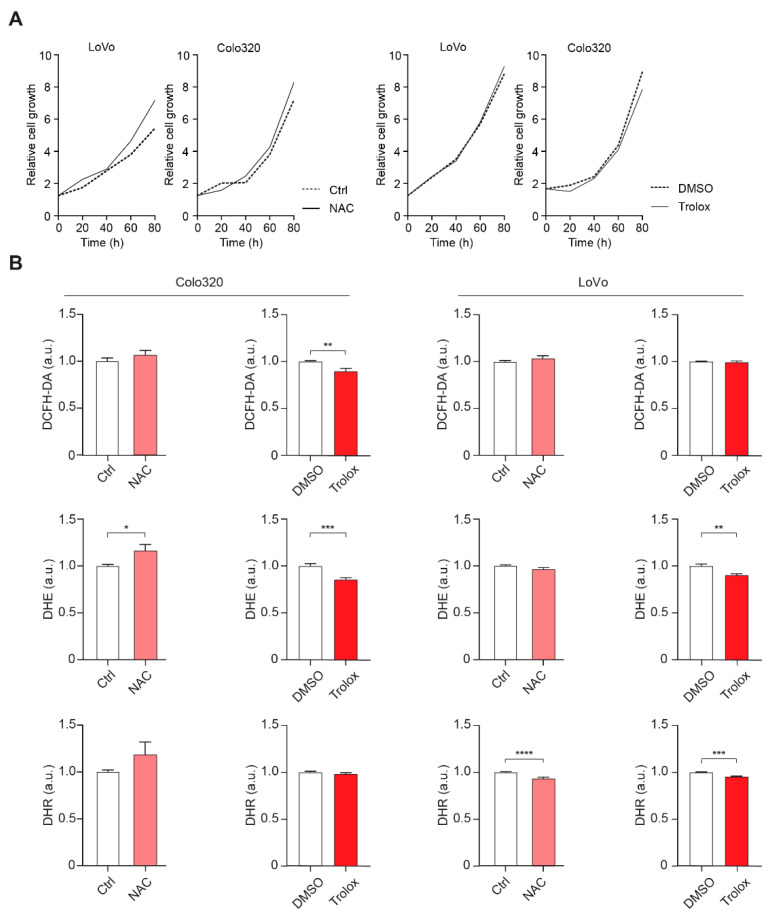
Effects of NAC or Trolox on proliferation of human colon cancer cell lines. (**A**) Real-time analyses of proliferation of two human colon cancer cell lines, LoVo and Colo320, cultured in medium supplemented with 200 μM NAC or 20 μM Trolox compared to untreated control (NAC) or DMSO control (Trolox); data are means of six replicates per cell line. (**B**) Graphs showing relative intensity of DCFH-DA, DHR, and DHE probes in LoVo and Colo320 cells, cultured as described above (*n* = 6). DCFH-DA, dichloro-dihydro-fluorescein diacetate; DHR, dihydrorhodamine; DHE, dihydroethidium; a.u., arbitrary units; error bars indicate SEM; * *p* < 0.05, ** *p* < 0.01, *** *p* < 0.001, **** *p* < 0.0001.

We next investigated whether NAC or Trolox, a water-soluble VitE analogue, affected the growth of two human colorectal cancer cell lines, LoVo and Colo320. Both cell lines carry mutations in the APC gene, suggesting that they are relevant cell culture models for this study. None of the cell lines were growth stimulated by NAC or Trolox compared to controls (Figure 5A). ROS quantification with dichloro-dihydro-fluorescein diacetate (DCHF-DA), dihydroethidium (DHE), and dihydrorhodamine (DHR) probes confirmed that Trolox lowered ROS in both cell lines, whereas NAC produced mixed results (Figure 5B). Thus, lowered ROS levels following NAC or Trolox treatment had no effect on the growth of human colorectal cancer cell lines in vitro.

## 4. Discussion

Accumulating data suggest that dietary antioxidants accelerate the progression of RAS-driven lung and skin tumors, but whether other types of tumors are susceptible to antioxidants is not clear. Here, we show that NAC or VitE, mixed in the drinking water or chow diet, increased the size and grade of intestinal tumors in *APC^Min/+^* mice without affecting the initiation of new tumors. The study raises the possibility that dietary antioxidants can accelerate the progression of a wide range of solid tumors. Importantly, NAC accelerated tumor progression at plasma concentrations that were comparable to or below those measured in human subjects that were given prescription doses of NAC.

There was no difference in tumor multiplicity between the antioxidant-treated *APC^Min/+^* mice and controls, indicating that tumor initiation was not affected. However, the majority of tumors in *APC^Min/+^* mice are formed prenatally, and no tumors are formed after day 100 [32,33]. Thus, insufficient transfer of NAC or VitE across the placenta or during breastfeeding might affect the result. The frequent use of NAC or VitE to prevent oxidative stress-related phenotypes in prenatal mice suggests that placental transfer is not a problem [34,35]. Additionally, poor transfer of NAC or VitE during breastfeeding would not explain the complete lack of effect on tumor multiplicity since the majority of tumors are initiated before birth.

Supplementation with NAC or VitE increased the size of tumors at week 12 and the numbers of tumors with high-grade dysplasia at week 19, i.e., phenotypes that are associated with malignancy and poor prognosis in humans. *APC^Min/+^* mice succumb to anemia or intestinal intussusception before tumors invade or metastasize. Whether prolonged exposure to dietary antioxidants is sufficient to transform intestinal adenomas to invasive carcinomas is therefore an open question. However, similar doses of NAC or VitE increased the metastasis of cutaneous melanomas [5] and non-small-cell lung cancer tumors [8] in genetic mouse models, and the tissue invasion of intravenously injected patient-derived melanoma cells [6]. Studies of colorectal cancer models that permit longer survival and metastasis are therefore warranted.

We observed increased tumor cell proliferation in the antioxidant-treated *APC^Min/+^* mice at 19 weeks of age, suggesting a potential mechanism behind the increased tumor size. However, there was no correlation in time between the effects on tumor size at 12 weeks of age and tumor cell proliferation at 19 weeks of age. Moreover, NAC or Trolox did not stimulate the growth of human colorectal cancer cell lines in a cell-autonomous manner. The results raise the possibility that intestinal tumors in *APC^Min/+^* mice are indirectly affected by antioxidants. Possible mediators include the microbiota, the immune system or stromal cells. Mechanistic studies on tumor formation in *APC^Min/+^* mice treated with low concentrations of NAC or VitE should be prioritized.

Redox signaling in colorectal/intestinal cancer progression is complex, as studies have found that endogenous antioxidants (NRF2 and GPX2) can support colorectal cancer progression [36,37]. In addition, ROS can inhibit the growth of colorectal cancer cells through the modulation of the activity of the redox-sensitive transcription factor AP-1 and, in turn, decrease COX-2 and VEGF expression [38,39,40]. Supplementation with antioxidants could lead to an increase in COX-2 and VEGF expression. On the other hand, however, the loss of APC itself can induce the production of ROS [41]. Studies have demonstrated that superoxide production by RAC1-dependent NADPH oxidase is critical for WNT signaling [42,43] and necessary for tumor formation after APC loss [31,32,33]. Superoxide deactivates nucleoredoxin and the destruction complex, and promotes the nuclear translocation of β-catenin and activation of β-catenin target genes [42,43]. In line with this, NAC treatment reduced tumor formation and extended the survival of APC^fl/fl^ Lgr5^GFP-CREER^ RAC1^fl/fl^ mice in which APC was conditionally deleted in crypt stem cells upon tamoxifen treatment [44]. Why supplementation with NAC or VitE promoted progression in our study is not clear. However, it has been suggested that ROS exist in two separate pools, either proliferative ROS produced by RAC1 or damaging ROS produced by the mitochondria, and that NAC at a low concentration neutralizes damaging ROS but not proliferative ROS [45]. Indeed, the NAC concentration used in our study was 5-fold lower compared to the concentrations used to curb the formation of APC-driven intestinal tumors in APC^fl/fl^ Lgr5^GFP-CREER^ RAC1^fl/fl^ mice [44].

The possibility that high and low concentrations of NAC might have different effects on intestinal tumorigenesis is of considerable interest. The estimated daily intake of NAC in our study equals 13 mg/kg of body weight in humans, after adjusting for body surface area (see methods). The prescription doses of NAC for long-term use range between 200 and 1200 mg (3–20 mg/kg of body weight), suggesting that our dose is comparable to those prescribed to humans. However, the bioavailability of drugs is governed by parameters such as basal metabolism, blood volume, circulating plasma proteins, renal function, and caloric expenditure, making drug-dose conversions between species challenging. Importantly, the plasma concentration of NAC in our study was 1 μM in the samples obtained during the light hours (Figure 1D). Since mice ingest two thirds of their total food and water (and NAC) during the dark hours [46,47,48], the peak concentration was likely higher. Peak concentrations in human volunteers were 4.8 μM after 600 mg of NAC [49] and 16 μM after 600 mg of NAC every 12 h for two weeks [30]. Thus, the concentration of bioavailable NAC in the *APC^Min/+^* mice was comparable to that in humans ingesting prescription doses of NAC.

## 5. Conclusions

In summary, supplementation with the antioxidants NAC or VitE accelerated the progression of intestinal tumors in *APC^Min/+^* mice without affecting the initiation of new tumors. Whether dietary or pharmacological antioxidants can convert benign tumors into carcinomas is an important question that requires further investigation; however, the results warrant caution against the use of antioxidant supplements in patients with familial adenomatous polyposis who develop great numbers of benign polyps from a young age.

## Data Availability

Data available on request.
